# UBM-guided scleral buckling for Schwartz-Matsuo syndrome with tear of nonpigmented epithelium of the ciliary body: a case report

**DOI:** 10.1186/s12886-021-01809-6

**Published:** 2021-01-19

**Authors:** Yao Wang, Zhongli Hu, Yifan Jiang, Huan Liu, Xiaoyun Fang

**Affiliations:** 1 Eye Center, Second Affiliated Hospital, School of Medicine, Zhejiang University, No. 88 Jiefang Rd, Hangzhou, 310009 China; 2 Department of Ophthalmology, Zhuji People’s Hospital of Zhejiang Province, 311800 Zhuji, China; 3 Department of Ophthalmology, People’s Hospital of Kaihua, Zhejiang Province, 324300 Kaihua, China; 4 Department of Ophthalmology, the First People’s Hospital of Lin’an District, 311300 Hangzhou City, Zhejiang Province China

**Keywords:** Schwartz‐matsuo syndrome, Rhegmatogenous retinal detachment, Tear of the nonpigmented epithelium of the ciliary body, Ultrasound biomicroscopy, Case report

## Abstract

**Background:**

Tears in Schwartz-Matsuo syndrome are generally confirmed by preoperative ophthalmoscopic examination. A case of Schwartz-Matsuo syndrome with a tear detected by ultrasound biomicroscopy (UBM) and treated by UBM-guided scleral buckling was reported, and its mechanism was analysed.

**Case presentation:**

A 40-year-old Chinese man presented with blurry vision and intermittent eye pain in his left eye for three days. The visual acuity of the left eye decreased from 20/20 to 20/40, and the intraocular pressure (IOP) fluctuated dramatically from 24.0 mmHg to 56.7 mmHg at the first visit. Gonioscopy revealed that the chamber angle remained open. A macula-involving inferior retinal detachment extending from 4:30 to 9:30 with no obvious causative break was observed through ophthalmoscopic examination. However, a single small tear was detected at the nonpigmented epithelium of pars plana of the ciliary body at approximately 7–8 o’clock by UBM. The loss of photoreceptor outer segments and ellipsoid zone and the existence of macular microcysts in the inner and outer nuclear layers were observed in the detached macula by optical coherence tomography. Then, he underwent successful scleral buckling guided by UBM. Three months later, the retina was flat with normal IOP, and the best corrected visual acuity of his left eye gradually improved to 20/25. UBM confirmed the closure of the tear.

**Conclusions:**

Tear of the nonpigmented epithelium of the ciliary body is a rare condition associated with Schwartz-Matsuo syndrome. UBM plays a key role in detecting occult tears of the nonpigmented epithelium of the ciliary body, guiding scleral buckling surgery, and observing the closure of the tear postoperatively.

## Background

Schwartz-Matsuo syndrome, which is also known as photoreceptor outer segment glaucoma, is characterized by increased intraocular pressure (IOP) with obvious fluctuation, cells in the anterior chamber (AC), and rhegmatogenous retinal detachment (RRD) [1]. In 1973, Schwartz et al. [2] first described 11 eyes with coexistence of RRD, elevated IOP, and aqueous cells. The high IOP typically normalized, and the aqueous cells disappeared quickly after retinal detachment repair. Photoreceptor outer segments (POS) in the aqueous humor were detected by electron microscopy by Matsuo et al. [3] in 1986. He suspected that these POS blocked the trabecular meshwork, affected the flow of aqueous humor, and eventually resulted in an increased IOP. Further studies confirmed that POS were directly detected in the aqueous humor or in the trabecular meshwork or contained in pigmented epitheloid cells in the aqueous humor [4–7]. Tears inducing retinal detachment are frequently located around the vitreous base in Schwartz-Matsuo syndrome, such as the oral dialyses and the nonpigmented epithelium of the ciliary body. We report a case of Schwartz-Matsuo syndrome caused by a tear of nonpigmented epithelium of the pars plana and highlight the key role of ultrasound biomicroscopy (UBM) in the detection of the tear, surgery planning and evaluation of tear closure.

## Case presentation

A 40-year-old Chinese man presented with complaints of blurry vision and intermittent eye pain in the left eye for three days. He denied a history of glaucoma, high myopia, blunt ocular trauma or any previous ocular surgery, and there was no known family history of significant ocular disease. However, the patient was skilled in badminton and had a history of dermatitis.

**Fig. 1:**
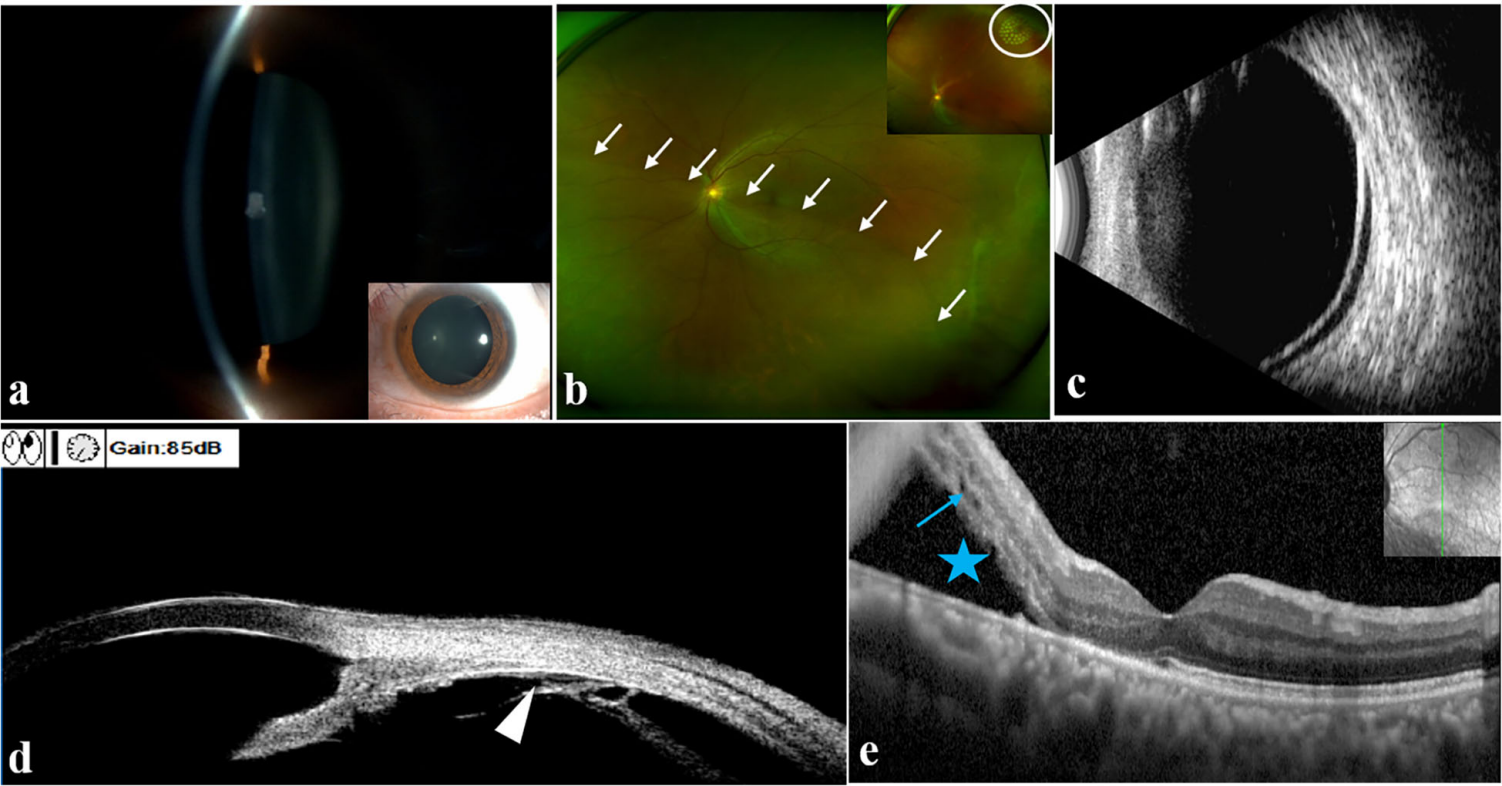
Images at the first visit preoperatively. **a** Anterior segment photograph revealed absence of aqueous cells, keratic precipitates, conjunctival hyperemia, and posterior synechiae in the left eye. **b** Optos color photograph noted inferior macula-involving retinal detachment extending from 4:30 to 9:30 (white arrow) and a retinal hole without surrounding SRF at 1 o’clock (white circle). **c** B-scan ultrasonography revealed extensive retinal detachment. **d** UBM revealed a single small tear of the nonpigmented epithelium of the pars plana of the ciliary body at 7–8 o’clock (white triangle). **e** OCT observed the loss of POS and ellipsoid zone (blue star) in the detached macula, and some macular microcysts (blue arrow) in the inner and outer nuclear layers

On presentation, visual acuity (VA) was 20/40 in the left eye and 20/20 in the right eye. Gonioscopy revealed the open chamber angle with regular iris insertions, and trabecular meshwork pigmentation in the left eye was slightly greater than that in the right eye. The anterior segment of the left eye represented an absence of aqueous cells, keratic precipitates, conjunctival hyperemia and posterior synechiae (Fig. 1a). Dilated ophthalmoscopic examination in the left eye revealed macula-involving retinal detachment extending from 4:30 to 9:30 and a cup-to-disc ratio of 0.4 (Fig. 1b and c). A retinal hole with no surrounding fluid was observed superiorly at 1 o’clock, and it did not communicate with the inferior RRD. No other obvious tear was found at the retinal detachment area through a detailed three-mirror lens examination as well as superfield condensing lens and widefield retina laser lens examinations. However, a single small tear of the nonpigmented epithelium of the pars plana of the ciliary body at 7–8 o’clock was detected by UBM, which was located approximately 3.59 mm from the limbus (Fig. 1d). The tear was definitely related to retinal detachment. The loss of the POS and ellipsoid zone in the detached macula and the existence of microcysts in the paracentral retina at the level of the inner and outer nuclear layers were observed by optical coherence tomography (OCT) (Spectralis OCT, Heidelberg, Germany) (Fig. 1e). Typically, the IOP of the left eye exhibited surprising fluctuations in the daytime at the first visit. The IOP was 24 mmHg in the left eye and 14.7 mmHg in the right eye in the morning, 31.0 mmHg in the left eye and 17.0 mmHg in the right eye at noon and eventually increased to 56.7 mmHg in the left eye with remarkable corneal epithelium oedema and 20.7 mmHg in the right eye in the afternoon. Based on the tear in the pars plana epithelium detected by UBM and the typical fluctuation of elevated IOP, a clinical diagnosis of Schwartz-Matsuo syndrome was confirmed.

The IOP became normal the day after medication with topical IOP-lowering agents (brimonidine, caroteol 2 %, brizolamide 1 %) and mannitol injection. Laser photocoagulation was performed to close the retinal hole without surrounding SRF at 1 o’clock (Fig. 1b). Aqueous humor was collected for transmission electron microscopy examination; unfortunately, POS were not detected. The IOP increased to 37.5 mmHg again the day after AC paracentesis.

**Fig. 2:**
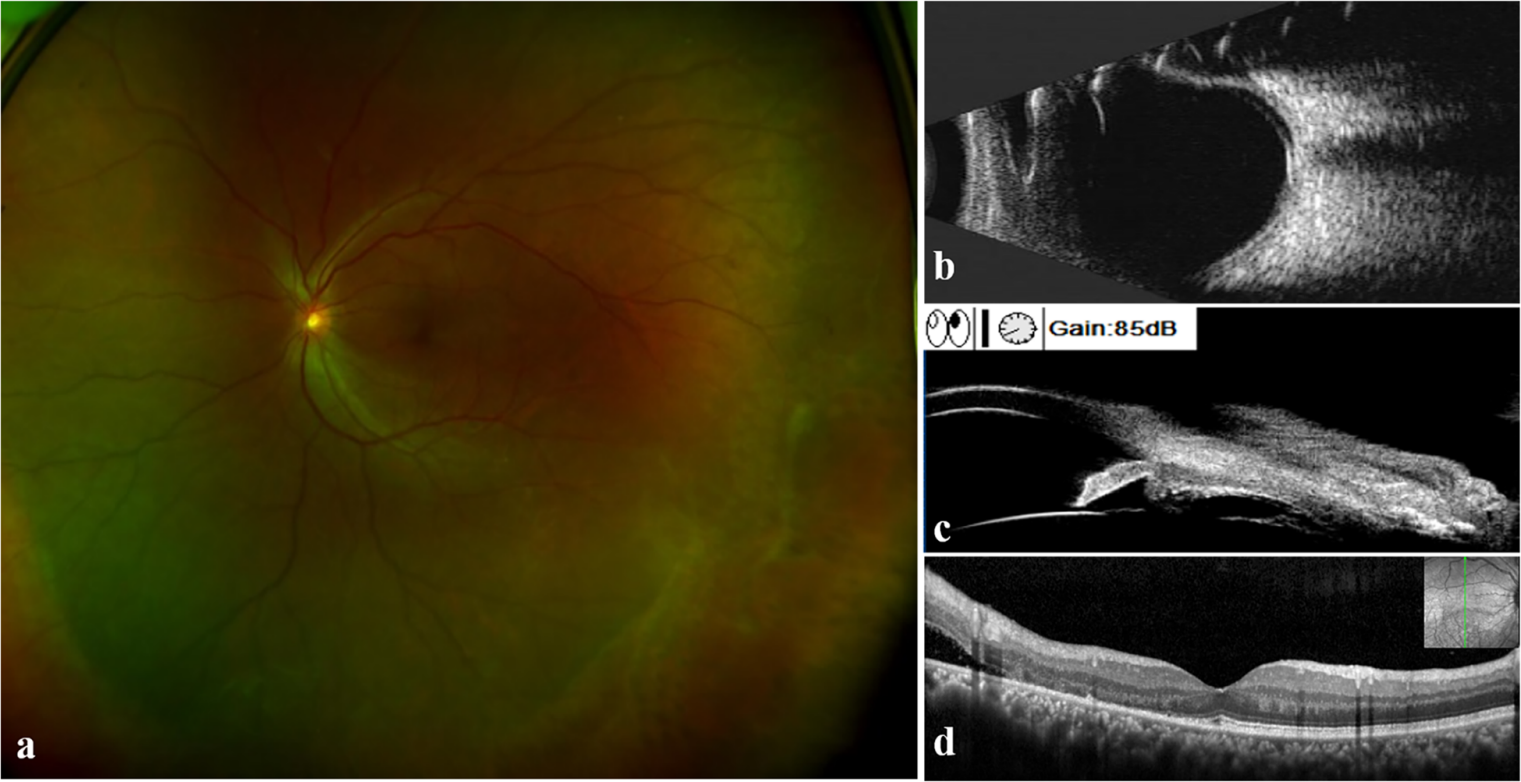
Optos color photograph **(a)**, B-scan ultrasonography **(b)**, UBM **(c)** and OCT **(d)** revealed that most of the retina was reattached and the SRF was obviously absorbed one week postoperatively

**Fig. 3:**
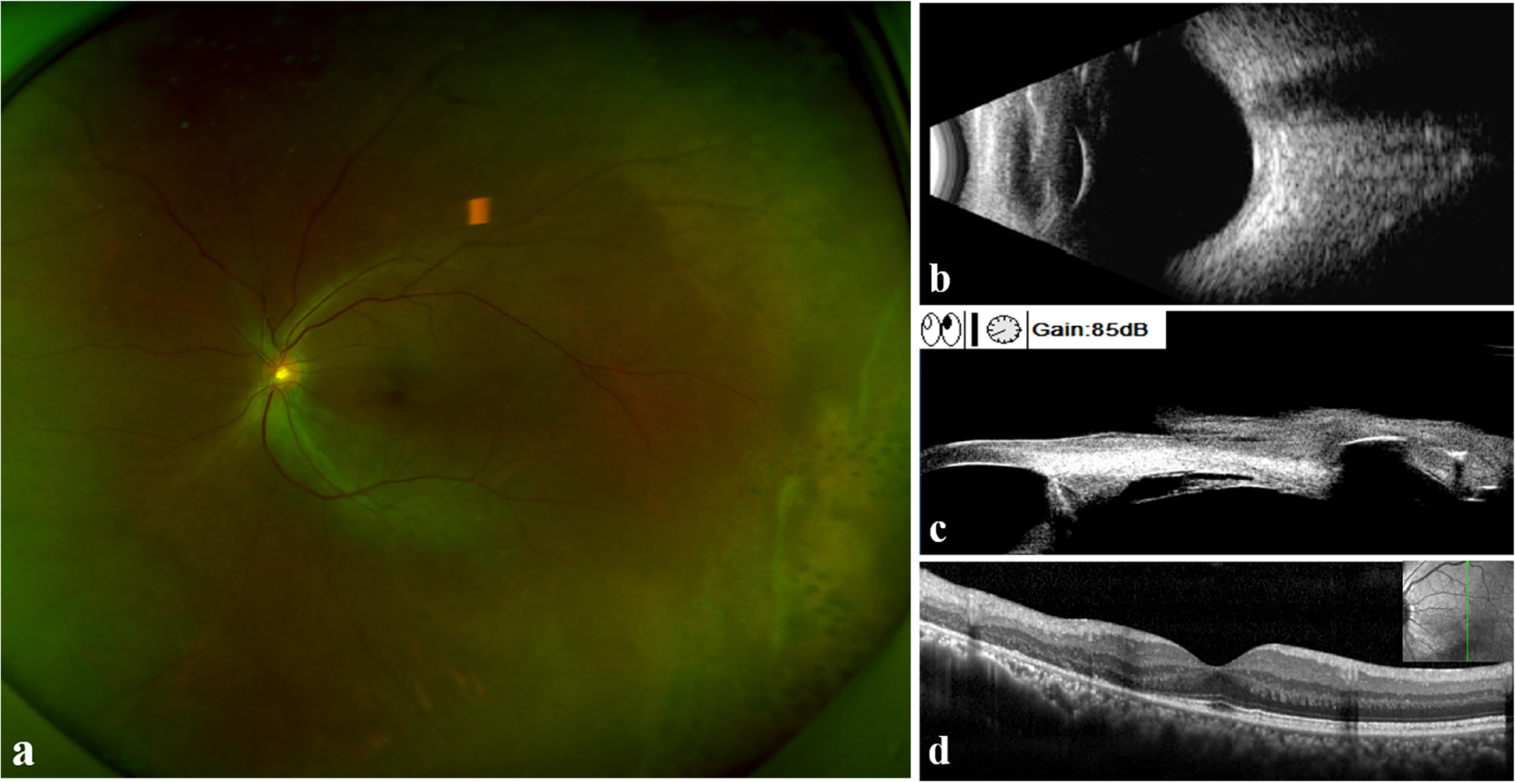
Optos color photograph **(a)**, B-scan ultrasonography **(b)** and OCT **(d)** proved that the retina remained attached and the SRF was entirely absorbed; UBM **(c)** revealed the tear was closed three months postoperatively

One week later, there was no obvious change in RRD, and IOP was 31 mmHg. The patient underwent a scleral buckling surgery. However, no definite causative tear at the retina or ciliary body was observed through intraoperative examination using indirect ophthalmoscope or surgical microscope with scleral indentation. Under the guidance of UBM, external cryocoagulation was applied at 7–8 o’clock of the pars plana site to cover the suspicious tear. A circumferential silicon sponge was applied at 6–9 o’clock at the oral serrata without subretinal fluid (SRF) drainage. On the third postoperative day, the IOP of the left eye decreased to 15.5 mmHg, and SRF was reduced. After one week, the IOP was 18.5 mmHg, and the VA was 20/40 in the left eye. Along with the obviously absorbed SRF, most of the retina was reattached (Fig. 2). Brimonidine and brizolamide 1 % were stopped, and only carteolol 2 % was used twice a day. Three months later, the best corrected visual acuity was 20/25 with refractive errors of -1.00/-0.75 × 139. The IOP was stable at 14.5 mmHg without any IOP-lowering agent. OCT and B-scan ultrasonography proved that the whole retina was attached, and UBM revealed that the tear was closed (Fig. 3).

## Discussion and conclusions

RRD is typically associated with decreased IOP as a result of increased outflow of aqueous fluid through exposed retinal pigment epithelium. Schwartz-Matsuo syndrome is characterized by elevated IOP with marked fluctuation, aqueous cells and RRD. Abnormal IOP and aqueous cells did not respond to anti-inflammatory drugs. Aqueous cells were recognized as POS rather than inflammatory cells under transmission electron microscopy by Matsuo et al. [3].

The tears are often small, and 87 % of tears occur in the ora serrata or the nonpigmented epithelium of the pars plana or pars plicata of the ciliary body in Schwartz-Matsuo syndrome [8, 9]. The tears of the nonpigmented epithelium of the ciliary body were mostly located in the superotemporal quadrant followed by the inferotemporal and inferonasal quadrants [10]. Small tears in the nonpigmented ciliary epithelium are not easily observed through routine examination. Slit-lamp biomicroscopy and binocular indirect ophthalmoscopy with scleral depression are essential for the peripheral fundal examination and pars plana examination preoperatively. In this case, no definite causative tear associated with inferior retinal detachment was identified through ophthalmoscopic examinations preoperatively. Nevertheless, UBM detected a small tear of the nonpigmented epithelium of the pars plana of the ciliary body at 7–8 o’clock associated with the inferior retinal detachment area. This crucial finding validated the clinical diagnosis of Schwartz-Matsuo syndrome, and the diagnosis of exudative retinal detachment was excluded. Furthermore, given that no definite tear could be confirmed through intraoperative ophthalmoscopic examination with scleral indentation, scleral buckling was performed under the guidance of UBM and ultimately succeeded. UBM also detected closure of the break in the pars plana epithelium three months postoperatively. In this case, UBM played a key role throughout the overall duration of treatment, including the diagnosis, guidance of surgery and follow-up of tear closure. UBM is a noninvasive and painless high-resolution ultrasound image diagnosis system for ophthalmology [11–13]. In addition to imaging of the anterior segment, UBM is useful for visualizing the pars plana, peripheral retina and anterior choroid regardless of corneal opacity [14, 15]. These features make up for the shortcomings of other ophthalmic examinations, such as gonioscopy and ordinary ultrasonic examinations. For the treatment of Schwartz-Matsuo syndrome, UBM could be a useful alternative for the location and monitoring of occult tears in the nonpigmented ciliary epithelium.

The tears in the pars plana epithelium occurred anterior to the vitreous base and established direct communication between the subretinal space and aqueous humor [10]. POS obstruct aqueous outflow at the trabecular meshwork and induce high intraocular pressure [1]. Unfortunately, POS were not detected by transmission electron microscopy in the aqueous humor in our case. Since no aqueous cells were observed preoperatively, the amount of POS in the aqueous humor sample was presumably too small to be detected. Matsuo et el. [10] hypothesised that when the number of aqueous cells was below level 1 + in the Hogan grading system, centrifuged cells would be difficult to find.

The IOP of the patient in our case fluctuated dramatically from 24.0 mmHg to 56.7 mmHg at the first visit and returned to normal after scleral buckling surgery in the absence of an IOP-lowering agent, which was consistent with the reports of Matsuo and Etheridge et al. [3, 5]. In some reported cases, although the IOP was not beyond the normal value, it had already presented remarkable fluctuation [10]. Matsushita et al. [16] concluded that IOP elevation differed among the patients with the highest level of 8 kPa (1 kPa = 7.5 mmHg). IOP fluctuated greatly in relation to the physiological rhythm and individual baseline. The outflow facility coefficient decreased with increasing IOP and returned to normal following decreasing IOP.

OCT confirmed the presence of macula-involving retinal detachment rather than retinoschisis. It is worth noting that adjacent loss of the POS and ellipsoid zone and the existence of microcysts in the inner and outer nuclear layers were observed at the detached retina, which proved chronic retinal detachment [5, 17]. OCT features might provide additional evidence of POS loss and entrance to the AC. The ellipsoid zone was still deficient, and the microcysts disappeared after retinal reattachment. Chen et al. [17] suggested that the microcysts in secondary glaucoma from Schwartz-Matsuo syndrome were due to severe ganglion cell loss. The vitreous and intact hyaloid face provided stable support for the macula, which prevented collapse after the loss of the inner nuclear layer and formed these macular microcysts.

A history of blunt ocular trauma is a risk factor for Schwartz-Matsuo syndrome, which dates back to several months or even years before those symptoms appear [18]. Our patient was good at sports, especially badminton. Although he denied a recent trauma of the left eye, a history of blunt ocular trauma could be suspected. In addition, the patient was diagnosed with dermatitis by the dermatologist, which might provide an additional clue for our diagnosis. Retinal detachment with atopic dermatitis is often related to oral dialyses or tears of the nonpigmented ciliary epithelium. One mechanical explanation is that head banging and face slapping due to severe itching may cause retinal trauma. The other endogenous assumption is that the retina, ciliary epithelium and vitreous may share an identical congenital disorder with the skin [19, 20].

Our patient suffered from a macula-involving RRD caused by a small tear of the nonpigmented epithelium of the pars plana of the ciliary body. The IOP was elevated with characteristic fluctuations in the left eye and returned to normal after successful scleral buckling surgery. These findings conform to the clinical manifestations of Schwartz-Matsuo syndrome. It is worth mentioning that no definite causative tear was confirmed through ophthalmoscopic examination, and tears of ciliary epithelium were detected by UBM, which also successfully guided scleral buckling surgery. To our knowledge, this study is the first report on UBM-guided scleral buckling for Schwartz-Matsuo syndrome. UBM played a key role in the tear location preoperatively, surgery planning and evaluation of tear closure postoperatively.

## Data Availability

The datasets used and analysed during the current study are available from the corresponding author on reasonable request.

## Abbreviations

IOP: Intraocular pressure
RRD: Rhegmatogenous retinal detachment
AC: Anterior chamber
POS: Photoreceptor outer segments
VA: Visual acuity
UBM: Ultrasound biomicroscopy
OCT: Optical coherence tomography
